# MXene-Graphene Composites: A Perspective on Biomedical Potentials

**DOI:** 10.1007/s40820-022-00880-y

**Published:** 2022-06-14

**Authors:** Ebrahim Mostafavi, Siavash Iravani

**Affiliations:** 1 Stanford Cardiovascular Institute, Stanford University School of Medicine, Stanford, CA 94305 USA; 2 Department of Medicine, Stanford University School of Medicine, Stanford, CA 94305 USA; 3 Faculty of Pharmacy and Pharmaceutical Sciences, Isfahan University of Medical Sciences, Isfahan, Iran

**Keywords:** Graphene, MXene, Composites, Hybrid structures, Biocompatibility, Cancer theranostics, Biomedical engineering

## Abstract

MXenes, transition metal carbides and nitrides with graphene-like structures, have received considerable attention since their first discovery. On the other hand, Graphene has been extensively used in biomedical and medicinal applications. MXene and graphene, both as promising candidates of two-dimensional materials, have shown to possess high potential in future biomedical applications due to their unique physicochemical properties such as superior electrical conductivity, high biocompatibility, large surface area, optical and magnetic features, and extraordinary thermal and mechanical properties. These special structural, functional, and biological characteristics suggest that the hybrid/composite structure of MXene and graphene would be able to meet many unmet needs in different fields; particularly in medicine and biomedical engineering, where high-performance mechanical, electrical, thermal, magnetic, and optical requirements are necessary. However, the hybridization and surface functionalization should be further explored to obtain biocompatible composites/platforms with unique physicochemical properties, high stability, and multifunctionality. In addition, toxicological and long-term biosafety assessments and clinical translation evaluations should be given high priority in research. Although very limited studies have revealed the excellent potentials of MXene/graphene in biomedicine, the next steps should be toward the extensive research and detailed analysis in optimizing the properties and improving their functionality with a clinical and industrial outlook. Herein, different synthesis/fabrication methods and performances of MXene/graphene composites are discussed for potential biomedical applications. The potential toxicological effects of these composites on human cells and tissues are also covered, and future perspectives toward more successful translational applications are presented. The current state-of-the-art biotechnological advances in the use of MXene-Graphene composites, as well as their developmental challenges and future prospects are also deliberated. Due to the superior properties and multifunctionality of MXene-graphene composites, these hybrid structures can open up considerable new horizons in future of healthcare and medicine.

## Highlights

- MXene/graphene composites possess high potential in future biomedical applications.
- The hybridization and surface functionalization of MXene-graphene composites should be further explored to improve the biocompatibility, high stability, and multifunctionality.
- The synthesis methods, performances, potential toxicologies, as well as future perspectives of MXene/graphene composites are discussed.

## Graphical abstract

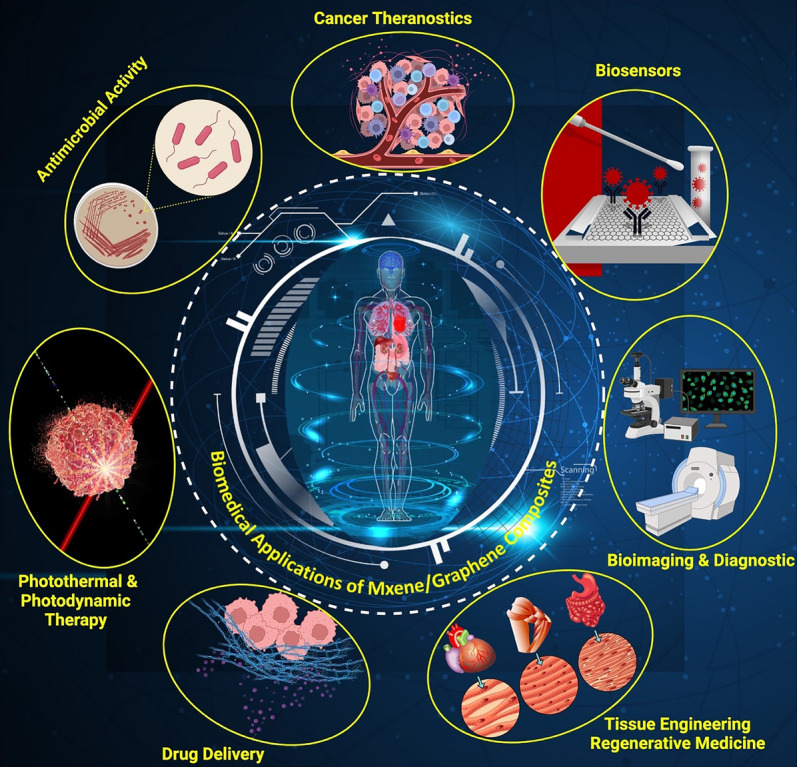

## Introduction

**Fig. 1:**
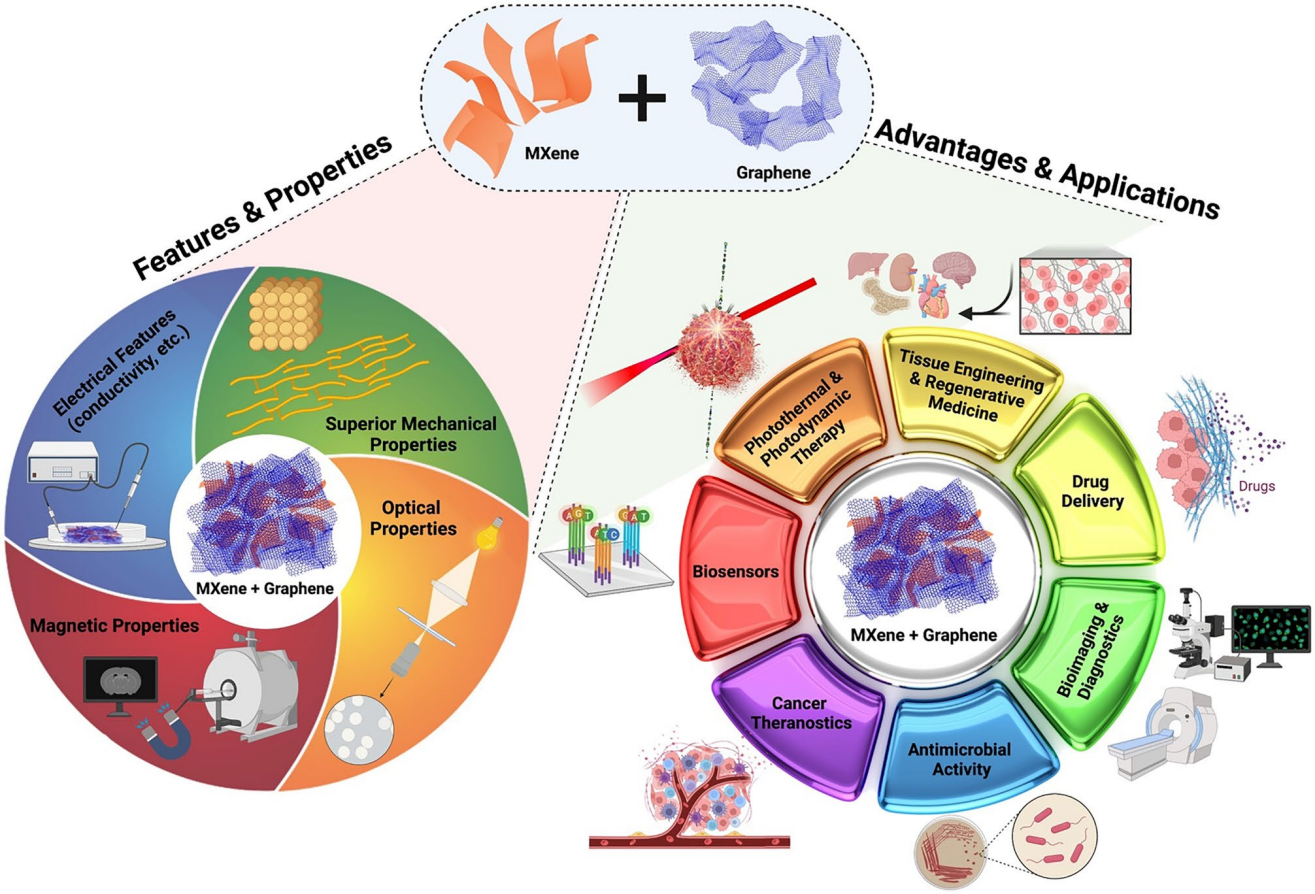
MXene-graphene hybrids with fascinating physicochemical properties/features can be considered as promising candidates for biomedical explorations

Today, with extensive advances in designing intelligent (nano)structures with the purposes of targeted delivery/therapy and diagnosis with high accuracy and efficacy, the hybridization of materials has been focused by researchers [1–3]. For instance, Tu et al. [4] introduced three-dimensional (3D) microflowers constructed from MXene/boron carbon nitride hybrids for wearable all-solid-state flexible micro-supercapacitors with high power density and large scalability [4]. MXenes can be hybridized to improve their features or attain new properties and multiple functionalities. MXene-based composites have promising applicability for high-performance energy-related devices and flexible bioelectronics [2, 5, 6]. MXene-based (nano)structures with high electrical conductivity, light-to-heat conversion, photocatalytic activity, and hydrophilicity have been broadly studied for manufacturing nanostructures with suitable multifunctionality [7–9]. Instead, graphene-based (nano)structures exhibited unique physicochemical properties such as pH sensitivity, stiffness, high electrical conductivity, large surface area, and mechanical strength [10, 11]; these materials with large surface area, high thermal/electrical conductivity, optical transmittance, electron mobility, and young modulus values have found their place in a variety of biomedical fields [12–14]. Since MXene nanosheet illustrated high efficiency as hybridization matrix over graphene, several MXenes/graphene hybrid composites have been designed with excellent structural robustness, conductivity, and flexibility properties as well as unique electrical/electrochemical and mechanical features (Fig. 1) [15–19]. These composites have shown an improved through-plane thermal conductivity, when they applied in polyethylene glycol matrix. The advanced electromagnetic interference (EMI)-shielding effectiveness of the designed composites reached ∼36 dB at the 2.5 mm thickness [20]. It was indicated that magnetic MXene (Ti_3_C_2_T_x_)-reduced graphene oxide aerogels anchored with magnetic nickel nano-chains exhibited suitable multifunctionality, hydrophobicity, and heat insulation activity [15]. In addition, MXene (Ti_3_C_2_T_x_)-graphene oxide hybrid foams were prepared via freeze-drying and reduction heat treatment techniques with enhanced electrical conductivity and superb EMI performance, which make them excellent candidates to be utilized in smart and next-generation of devices [21].

MXene-graphene hybrids with high conductivity, thermal stability, and excellent EMI have found their applications in designing novel supercapacitors and multifunctional sensors [22, 23]. 3D MXene (Ti_3_C_2_T_x_)-graphene hybrid aerogels with aligned cellular microstructures were prepared through hydrothermal assembly followed by directional-freezing and freeze-drying processes [24]. These porous materials with significantly conductive architectures (up to 1085 S m^–1^) exhibited superb electrical conductivity (695.9 S m^–1^) and EMI-shielding effectiveness (more than 50 dB in the X-band at a low MXene content of 0.74 vol.%) [24]. Additionally, the self-healing ability is one of the important properties that should be considered for manufacturing smart and long-life multifunctional devices based on sustainable technologies. As an example, self-healable MXene (Ti_3_C_2_T_x_)-graphene composite aerogel electrodes with high conductivity and large specific surface area were constructed utilizing self-healing polyurethane (outer shell) [25]. Likewise, biomimetic MXene-graphene oxide fibers designed by inspiring from the structure of wood exhibited significant tensile strength with high electrical conductivity, providing structures with unique morphologies and functionalities for high-value fabric-based applications [26]. Since, there are very limited studies around the biomedical applications of MXene-graphene hybrids, and there is still a gap in this field regarding the important challenges, optimization, and functionalization of them, particularly in bio- and nanomedicine; herein, we specifically discussed about the biomedical potentials of MXene-graphene hybrids with recent advancements and important challenging issues to motivate researchers for further explorations in this field of science.

## Synthesis Approaches

MXenes have been typically prepared *via* the selective removal of “A” layer from their MAX or non-MAX phase parents by acid etching, where A is generally group 13 or group 14 elements in the periodic table [27, 28]. Additionally, several top-down and bottom-up techniques have been introduced for synthesizing MXenes, including urea glass method [29], chemical vapor deposition [30], molten salt etching [31], hydrothermal fabrication [32], and electrochemical preparation [33]. Chemical vapor deposition and wet etching techniques have been widely reported for fabricating MXenes [34]. Notably, high-qualified MXenes with the presence of terminations were fabricated by applying different wet etching methods, causing to produce MXenes with basically hydrophilic nature [35]. On the other hand, graphene structures with desired size, purity, and crystallinity have been broadly fabricated by chemical vapor deposition, mechanical exfoliation from graphite, and reduction in graphene oxide *via* heating [36].

**Fig. 2:**
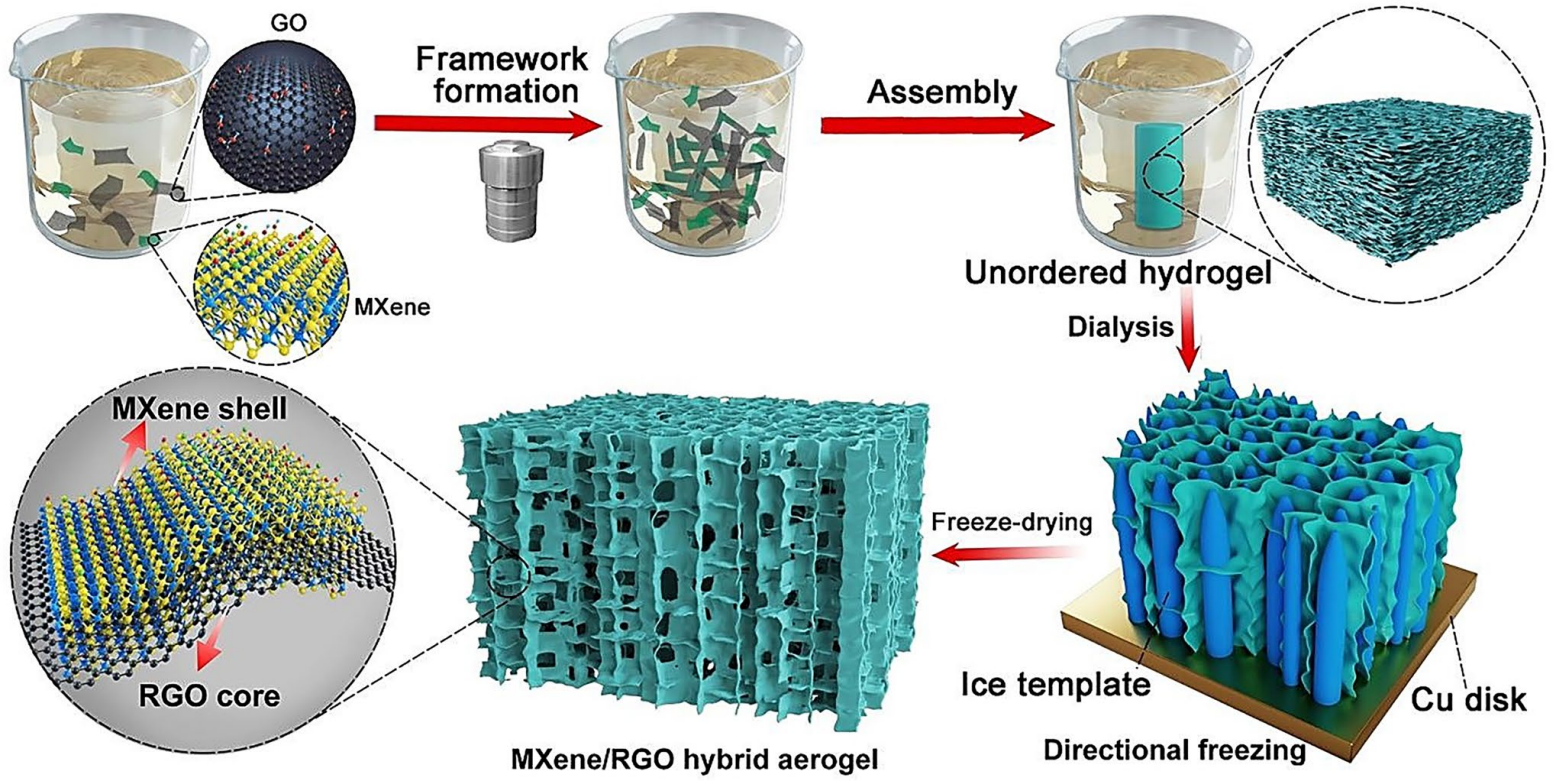
The preparative process of MXene-reduced graphene oxide (RGO) hybrid aerogels through GO-assisted hydrothermal assembly technique followed by directional-freezing and freeze-drying processes. Reproduced with permission from Ref. [24]. Copyright 2018 American Chemical Society

Various physical and chemical approaches have been reported for the synthesis of MXene/graphene composites, including mechanical mixing, self-assembly method, hydrothermal technique, heat treatment, and reagent reduction treatment. Among them, hydrothermal techniques have been widely applied to prepare composites. For instance, MXene (Ti_3_C_2_T_x_)/reduced graphene oxide structures were prepared at low temperature (65 °C) followed by a freeze-drying process. In the hydrothermal reaction, ascorbic acid was utilized to prevent MXene structures from being oxidized (Fig. 2) [24]. Additionally, 3D porous MXene (Ti_3_C_2_T_x_)/reduced graphene oxide aerogels were synthesized via a hydrothermal technique under a temperature of 95 °C. The designed electrodes from these hybrid aerogels exhibited high electrochemical activities, including significant capacity (~1270 mAh g^−1^ at 0.1C), enhanced cycling life (~500 cycles), and low capacity decay rate (~0.07% per cycle), with excellent areal capacity (5.27 mAh cm^−2^). Such composites with unique properties should be further explored for constructing intelligent devices with biomedical potentials [37].

**Fig. 3:**
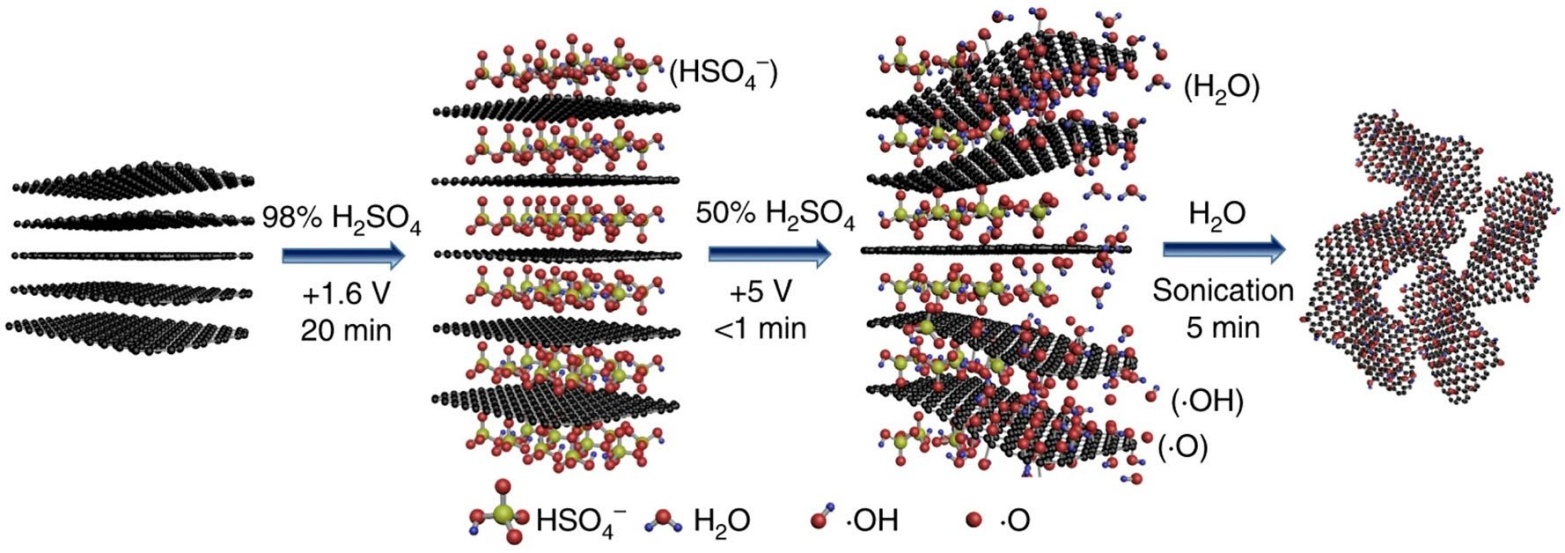
The preparative process of graphene oxide (GO) *via* the water electrolytic oxidation process. Reproduced with permission from Ref. [39]

Several methods have been reported for synthesizing MXenes and graphene based on green chemistry principles to avoid the utilization of toxic/harmful agents and laborious processes. However, greener methods for synthesis and functionalization are still in the infancy stages, and more elaborative studies should be planned to find low-cost, simple, up-scalable, and environmentally benign techniques for the synthesis of these structures. In one study, MXene nanosheets were synthesized using an electrochemical method without using dangerous acid/alkali etchants; although the prepared MXenes exhibited high stability and battery performance, it should be further explored for a variety of applications [38]. Greener method for fabricating graphene oxide sheets by water electrolytic oxidation of graphite was also reported (Fig. 3). The pre-intercalation of graphite could successfully inhibit the anodic electrocatalytic oxygen evolution reaction of water at high voltage to permit the ultrafast oxidation of graphene lattice within a few seconds [39]. Besides, porous graphene was eco-friendly synthesized via the combination of sodium citrate treatment, hydrothermal reduction, and lyophilization processes. These graphene structures were deployed for designing biosensors with high sensitivity and selectivity (the low limit of detection was ~83.0 nmol L^−1^) [40].

## Biomedical Prospects

MXenes and MXene-based (nano)structures possess abundant functional groups on their surfaces, offering modification/functionalization opportunities with flexibility. In addition to their high stability and hydrophilicity, they also contain complete metal atomic layers and tunable composition which make them attractive candidates for clinical and biomedical purposes [9]. For instance, smart nanoscale systems have been constructed by applying MXenes and graphene structures with cancer therapy/diagnosis and drug delivery potentials [41–44]. In one study, biocompatible MXene (Ti_2_N) quantum dot-based systems with high stability and targeting/selectivity properties were introduced with cancer photoacoustic imaging and photothermal therapy potentials [45]. Furthermore, biocompatible MXene (Ti_3_C_2_T_x_) structures with cellular uptake features exhibited high transferring potentials from vascular endothelial cells with localization, stability, and auto-fluorescence advantages at different emission-excitation wavelengths allowing post-transport examination and tracking [46]. On the other hand, graphene- and MXene-based (nano)structures have been deployed in constructing smart delivery systems for antiviral or antimicrobial drugs in addition to the production of antiviral/antimicrobial surface coatings and medical equipment (*e.g*., face masks) [47, 48]. Growth kinetics studies demonstrated that MXene nanosheets with sharp edges could directly and physically interact with the surface membrane of bacterial cells, causing the release of cellular materials from the cells [49]. It was revealed that graphene oxide derivatives could successfully obstruct the infection of HSV-1, mimicking the cell surface receptor heparan sulfate [50]. However, there are limited studies focused on MXene-based structures for detecting or inhibiting pathogenic viruses. As an example, MXenes (Ti_3_C_2_) could be applied for recognition of human papillomavirus (HPV) with high selectivity and fluorescence quenching ability to dye-labeled single-stranded DNA (ssDNA) as well as significant affinity for ssDNA and double-stranded DNA (dsDNA) [51]. Under the fluorescence quenching influence of the MXene nanosheets, ssDNA probe exhibited the minimal fluorescent emission, providing magnified fluorescent biosensor for specific recognition of HPV-18 (the low limit of detection was ~100 pM) [51].

Different types of MXene and graphene-based (nano)composites have been deployed for tissue engineering and regenerative medicine purposes, with efficient multifunctionality and good biocompatibility. Mi et al. [52] introduced 3D-printed tissue-engineered bone scaffolds using MXene (Ti_3_C_2_)-based structures to repair bone defects; MXene structures were incorporated into composite scaffolds constructed from hydroxyapatite and sodium alginate through extrusion-based 3D printing for bone regeneration. These scaffolds with uniform structures and macropore morphologies had significant mechanical strength with improved alkaline phosphatase performance, upregulated osteogenic gene expression, suitable biocompatibility, and stimulated mineralized-nodule generation/cell proliferation. They could efficiently promote the regeneration of bone (in vivo), providing great opportunities for bone healing [52]. Notably, MXene-based composites exhibited suitable hydrophilicity because of the presence of functional hydrophilic groups, providing microenvironment for growing bone marrow-derived mesenchymal stem cells [53]. They had good biocompatibility and improved cellular activity, and also could increase the differentiation of stem cells to osteoblasts [53]. MXene (Ti_3_C_2_) quantum dots with immunomodulatory effects have been explored for improving tissue repairing after injury. They selectively reduced the human CD4^+^IFN‐γ^+^ T‐lymphocytes activation and stimulated the expansion of immunosuppressive CD4^+^CD25^+^FoxP3^+^ regulatory T‐cells in a triggered lymphocyte population [54]. Biocompatible chitosan‐based hydrogels with thermo-sensitivity, conductivity, and injectability were produced using MXene quantum dots for stem cell and tissue repairing purposes [54]. Additionally, various composites of MXenes and graphene have been studied for their possible free-radical scavenging applications toward reactive oxygen stress and reactive nitrogen species. They have shown suitable antioxidant performances to protect the cells from oxidative damages, providing great opportunities for free-radical scavenging applications [55].

**Fig. 4:**
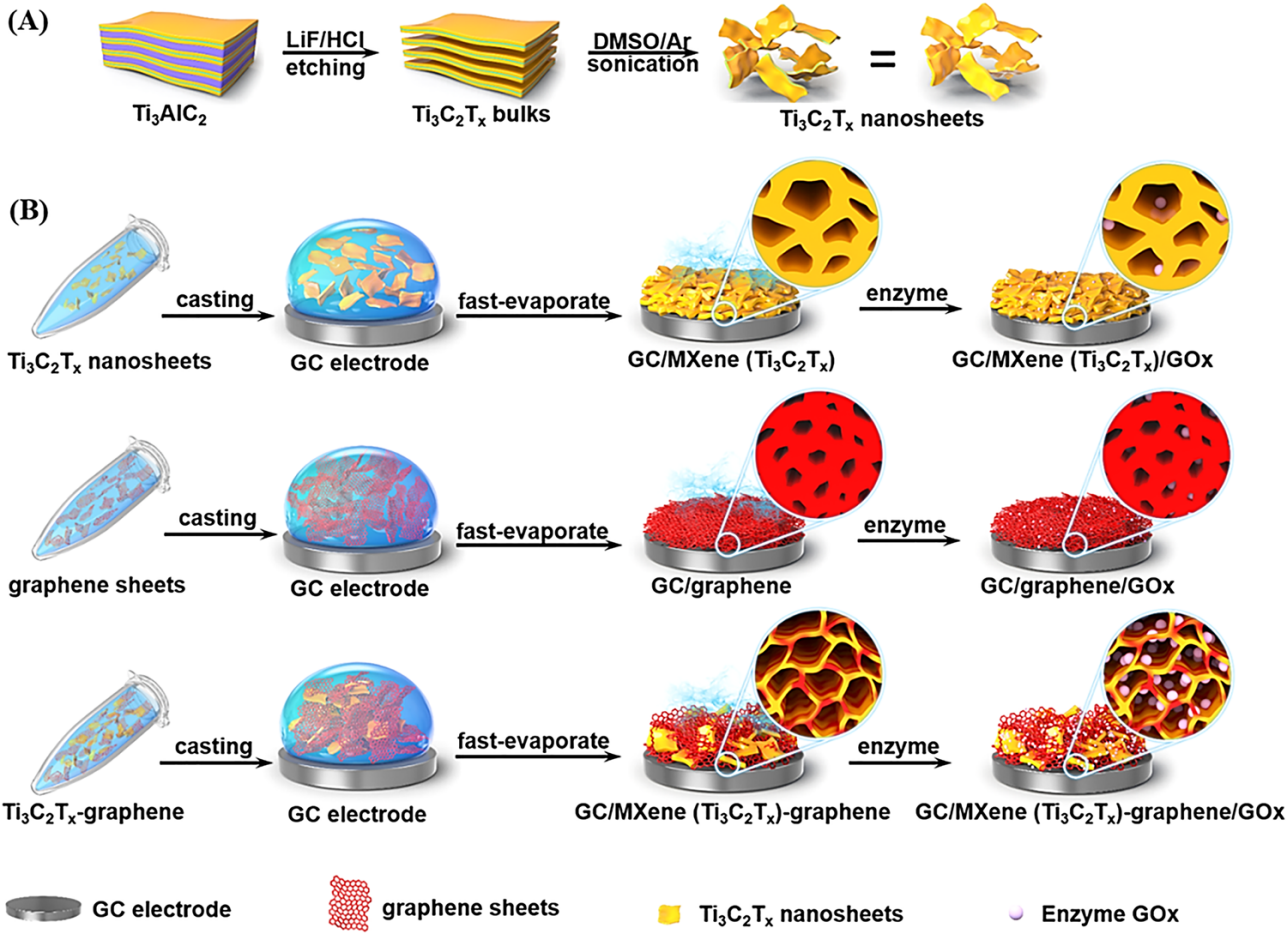
**A** The preparative processes of MXene nanosheets and **B** MXene-graphene hybrid films for the immobilization of enzymes with glucose biosensing application. LiF—Lithium fluoride; DMSO—Dimethyl sulfoxide; GC—Glassy carbon; GOx—Glucose oxidase. Reproduced with permission from Ref. [60]. Copyright 2019 American Chemical Society

The designed MXene-graphene hybrids have been applied as flexible supercapacitors, electrodes, ion batteries, and EMI-shielding [56–58]. However, there are some impressive explorations focused on their applications in (bio)sensing. For instance, MXene-graphene field-effect transistor sensors were designed for detecting influenza virus and coronavirus, with significant chemical sensitivity *via* antibody-antigen binding to obtain electrochemical signal transduction after the deposition of viruses onto the virus-sensing transduction material surface. The detection limit was as low as ~125 copies mL^-1^ for the influenza virus and 1 fg mL^-1^ for the recombinant 2019-nCoV spike protein [59]. Additionally, the incorporation of MXenes and graphene structures together can provide suitable porous materials with high binding capacity to enzymes with improved affinity and stability [60]. In one study, 3D porous MXene (Ti_3_C_2_T_x_)-graphene hybrid films were synthesized using a mixing-drying technique to produce biosensors for glucose detection (Fig. 4). Consequently, the designed biosensor demonstrated noticeable electrochemical catalytic performance toward glucose biosensing suitable for glucose assays in sera. After controlling MXene and graphene nanosheets ratio, the internal pore size could be optimized, affecting the immobilization of glucose oxide as well as glucose biosensing efficiency [60]. Besides, MXene (Ti_3_C_2_T_x_)/graphene/polydimethylsiloxane layered structures fabricated *via* vacuum filtration and pre-polymerization mainly contained two layers of MXenes (upper layer) and flexible graphene/polydimethylsiloxane composites (bottom layer). These composite films could be deployed in designing wearable strain sensors (especially for precise monitoring of full-range human motions) with a large range of linear response, as well as high sensitivity (low limit of detection was ~0.025%), linearity (R^2^ > 0.98), and cycling stability (more than 5,000 cycles) [61].

Layer-structured homogenous MXene (Ti_3_C_2_T_X_)-graphene oxide film-based sensors were designed with flexibility, conductivity, and cycling stability advantages [62]. One study designed an aerosol jet printed flexible bimodal sensor using graphene and MXene (Ti_3_C_2_T_x_) composites. The designed temperature sensor exhibited high sensitivity/accuracy and competitive thermos-power output (~53.6 μV/°C) with great flexibility/stability (negligible degradations after 1000 bending cycles), opening many opportunities for manufacturing multifunctional devices with biomedical potentials [1]. Notably, the d-spacing and oxygen groups were successfully controlled by MXene/graphene oxide composition ratio. These MXene-graphene composites exhibited long-term stability by suppressing MXene oxidation via the utilization of graphene oxide. These materials with unique features of linear sensitive response to humidity and high biocompatibility should be further explored in designing smart actuators as well as sensing and biology/health care devices (*e.g*., respiratory monitoring sensors) [62]. Furthermore, 3D aerogel-based piezoresistive sensors with superb linear sensitivity (331 kPa^−1^ from 0–500 Pa, 126 kPa^−1^ from 500 Pa–7.5 kPa) and high conductivity were designed utilizing MXene-reduced graphene oxide aerogels [63]. These sensors exhibited high performance and stability (even after 17,000 compression cycles) in addition to the fast response time (load 71 ms, recovery 15 ms) and low detection limit (1.25 Pa). They can be further evaluated for designing sensors with detection capabilities of heartbeat, breathing, and vocalization of the human body in real-time, showing their future applicability in flexible wearable electronic devices [63].

## Biocompatibility and Toxicity Issues

**Fig. 5:**
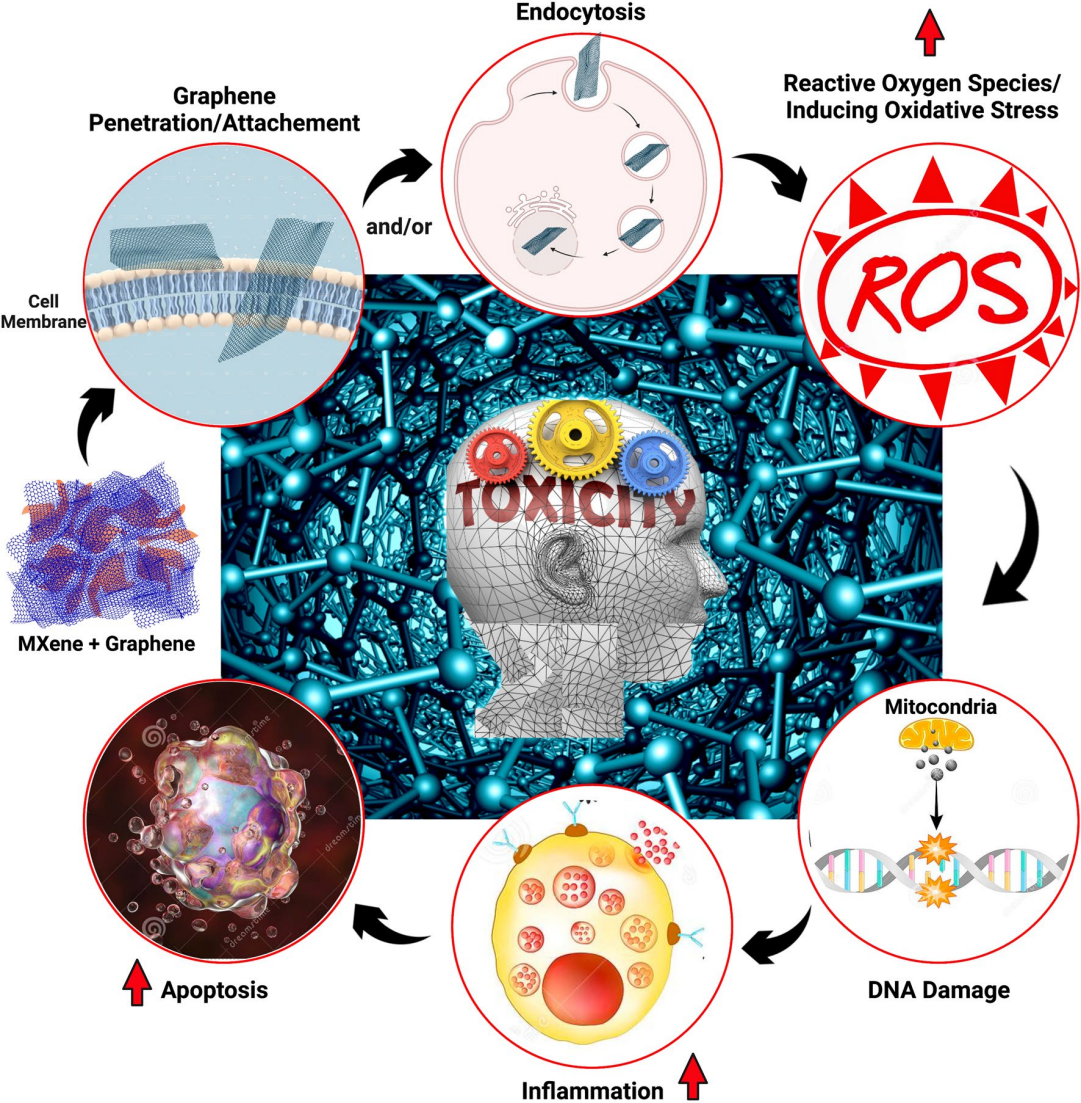
The potential mechanism of toxicity to human cells upon exposure to graphene-based materials

Graphene-based materials have been extensively used in various biomedical applications such as bioimaging, biosensing, theranostics, drug/gene delivery, antibacterials/antivirals, and tissue engineering applications. Therefore, for any effective and successful translation of these materials and becoming commercialized products, there should be a significant exposure of the human body to graphene-based materials, which makes it essential to evaluate the degree of biocompatibility and cytotoxicity of these materials to human cells. The potential cytotoxic effects of graphene-based materials on human cells mainly depend on their physicochemical characteristics, the nature of their interaction with cells, and their accumulation in specific tissues/organs [64]. The toxicological effects of graphene-MXene composites are also expected to be most affected by the presence of graphene and to further elaborate on these effects, a deep understanding of their cellular and molecular interactions with human cells/tissues/organs is required [65–68]. Figure 5 shows the potential mechanism of action encompassing hierarchical events that happen in human cells upon exposure to graphene-based materials.

For clinical translation of MXene- and graphene-based (nano)structures, future studies should be comprehensively focused on (eco)toxicological and cytotoxicity properties of these materials [69–72]. For instance, the biocompatibility of MXenes (Ti_3_C_2_T_x_) was analyzed for possible toxicity in a zebrafish embryo model (*in vivo* assessments) [73]. Accordingly, the zebrafish embryos could uptake MXenes with dose-dependent behavior, with the highest NOEC (no observed effect concentration) ≈50 μg mL^−1^, the lethal concentration 50 ≈257.46 μg mL^−1^, and LOEC (lowest observed effect concentration) ≈100 μg mL^−1^. Notably, no meaningful teratogenic influences could be detected in the examined model at 100 μg mL^−1^. After locomotion and neurotoxicity assessments, MXenes (50 μg mL^−1^) demonstrated no harmful influences on neuromuscular performances. Based on the results at concentrations below 100 μg mL^−1^, these MXene structures could be categorized as practically nontoxic based on the Acute Toxicity Rating Scale (ATRS) by the Fish and Wildlife Service [73]. By developing eco-friendly methods for the synthesis of MXenes and graphene materials, their biosafety features can be highly improved [73–78]. Also, surface functionalization of these structures by applying suitable bioactive and biocompatible agents can help to enhance their stability, pharmacokinetics, biocompatibility, and targeting properties, causing high specificity and reduced off-target effects [79]. Another crucial aspect that should be noted for their future clinical and biomedical applicability is the reduction in their stability due to the undesired events such as aggregations or accumulations, which can reduce their performances/functionalities and surface area [80, 81].

## Conclusions and Future Outlooks 

MXene-graphene hybrids have been explored due to their fascinating physicochemical properties, which make them attractive candidates for future biomedical explorations. Although MXenes-graphene nanomaterials have attracted wide attention in bio- and nanomedicine, their possible applications for drug delivery, cancer therapy, and theranostics are still uncertain concerning their biocompatibility and toxicity, lack of clinical assessments, and enough specificity/selectivity. Their biological sensing and imaging properties are typically restricted by their non-specific adsorption. MXenes with a larger interlayer spacing had higher specific surface area and additional exposed active sites. In this context, hybridization or surface modifications can remarkably improve multifunctionality and reduce the possible toxicity of these structures. Natural polymers (*e.g.*, cellulose or chitosan nanofibers) can be combined with MXenes and graphene structures to improve their biomedical applicability. Additionally, the commercialization and eco-friendly manufacturing of these structures should be comprehensively explored to find inexpensive and up-scalable strategies with higher safety profile. Optimization of reaction conditions, environmental stability, surface chemistry characterizations, nanotoxicological studies, systematic biocompatibility analyses (both *in vitro* and *in vivo*), and pre-/clinical assessments still need to be addressed. MXene structures with single-layer, fewer defects, and larger size had higher conductivity, illustrating that the synthesis conditions and their properties can significantly affect their quality and future applications; the intrinsic features of MXenes and graphene can be improved by controlling reaction mixture conditions (*e.g.*, pH and temperature), surface functional groups/terminations, and interlayer spacing.
